# Dietary inflammatory potential, genetic susceptibility, and systemic inflammation indices in relation to abdominal aortic aneurysm risk: a prospective cohort study

**DOI:** 10.3389/fnut.2025.1680225

**Published:** 2025-10-17

**Authors:** Duoliang Wei, Yongliang Zhong, Xinyi Liu, Rutao Guo, Yipeng Ge, Zhiyu Qiao, Junming Zhu

**Affiliations:** Department of Cardiovascular Surgery, Beijing Aortic Disease Center, Beijing Anzhen Hospital of Capital Medical University, Beijing, China

**Keywords:** abdominal aortic aneurysm, dietary inflammation, polygenic risk score, systemic inflammation response index, cohort study

## Abstract

**Background:**

Chronic inflammation and genetic susceptibility are important factors in abdominal aortic aneurysm (AAA) pathogenesis, yet evidence regarding the impact of dietary inflammation on AAA risk remains limited. This study aimed to investigate the association between dietary inflammatory potential, genetic susceptibility, and systemic inflammation in relation to AAA incidence.

**Methods:**

In this prospective cohort study, 142,862 participants from the UK Biobank were followed over an average of 13.8 years. Dietary inflammatory potential was assessed using the Energy-Adjusted Dietary Inflammatory Index (E-DII), while genetic susceptibility was quantified using polygenic risk scores (PRS) derived via PRS-CS methodology. Systemic inflammation indices, including the Systemic Immune-Inflammation Index and the Systemic Inflammation Response Index (SIRI), as well as nutritional and immunological status assessed by the Prognostic Nutritional Index and the Controlling Nutritional Status score, were also examined. In addition, the mediating roles of systemic inflammation indices were evaluated.

**Results:**

Higher E-DII scores were significantly associated with increased AAA risk (HR: 1.36, 95% CI: 1.09–1.71). Individuals with high PRS and high E-DII exhibited a markedly elevated AAA risk compared to those with low PRS and low E-DII (HR: 3.04, 95% CI: 2.21–4.79). SIRI mediated 9.16% (95% CI: 4.81%–17.90%) of the association between dietary inflammation and AAA.

**Conclusion:**

This study demonstrates that both dietary inflammatory potential and genetic susceptibility are associated with increased AAA risk, highlighting SIRI as a critical mediator. These findings suggest the potential utility of integrating dietary strategies, genetic screening, and inflammatory biomarkers into targeted AAA prevention programs.

## Introduction

Abdominal aortic aneurysm (AAA) is defined as a localized dilation of the abdominal aorta exceeding 3 cm or 50% of its normal diameter (1). It is often asymptomatic until life-threatening events such as expansion, dissection, or rupture occur (2). Ruptured AAAs are associated with high mortality, 65%–75% die before hospital arrival, and 35%–45% of those undergoing surgery still die perioperatively (3). Given its silent progression and fatal outcomes, early identification of risk factors and timely preventive strategies are crucial for reducing the burden and complications of AAA.

In recent years, a growing body of research has highlighted the protective role of healthy dietary patterns in the prevention of various cardiovascular diseases (4). However, evidence regarding the role of diet in the development of AAA remains limited. Chronic low-grade inflammation is recognized as a key contributor to AAA pathogenesis (5). The Energy-Adjusted Dietary Inflammatory Index (E-DII), a literature-derived score based on the pro- or anti-inflammatory properties of various nutrients, has been widely used to quantify the overall inflammatory potential of diet (6). Although higher E-DII scores have been linked to increased systemic inflammation and cardiovascular risk (7), the association between dietary inflammatory potential and the risk of AAA has not been fully elucidated.

In addition, composite blood-based markers such as the Systemic Immune-Inflammation Index (SII) and the Systemic Inflammation Response Index (SIRI)-which incorporate platelet, neutrophil, monocyte, and lymphocyte counts-have been proposed as integrative indicators of immune-inflammatory status (8). These markers have shown predictive potential in several vascular conditions and may offer insights into inflammatory pathways involved in AAA development (9). Similarly, the Prognostic Nutritional Index (PNI), derived from serum albumin and lymphocyte counts, has been widely used as a simple measure of nutritional and immunological status (10). The Controlling Nutritional Status (CONUT) score, based on serum albumin, total cholesterol, and lymphocyte counts, has also been validated as a comprehensive screening tool for nutritional risk and has demonstrated prognostic value across cardiovascular and surgical populations (11). Nevertheless, their mediating role between dietary inflammation and AAA risk remains largely unexplored. Furthermore, polygenic risk scores (PRS), derived from genome-wide association studies (GWAS), provide a quantitative measure of genetic susceptibility (12). Whether genetic predisposition, as measured by PRS, modifies the association between dietary inflammatory potential and AAA risk is still uncertain.

In this study, we aimed to evaluate the association between dietary inflammatory potential, as measured by the E-DII, and the risk of incident AAA in a large prospective cohort from the UK Biobank. We further assessed the joint effect of E-DII and polygenic risk scores on AAA risk. Finally, we investigated whether systemic inflammatory indices mediate the relationship between dietary inflammation and AAA development.

## Methods

### Study design and participants

This study utilized data from the UK Biobank, a large-scale prospective cohort study with over 500,000 participants. The individuals, aged 37–73, were recruited from 22 assessment centers across the United Kingdom between 2006 and 2010. During the initial enrollment, participants completed an extensive touchscreen questionnaire covering a wide range of health-related information, such as demographics, socioeconomic status, lifestyle habits, and medical history. Detailed information on the study’s design and data collection procedures can be found in existing publications (13).

A total of 210,965 participants were initially enrolled in this cohort study, all of whom completed at least one 24-h online dietary recall questionnaire at baseline. Participants were excluded if they refused follow-up, reported implausible energy intakes (<500 or >3,500 kcal/day for women; <800 or >4,200 kcal/day for men), were of non-white British ancestry, had a baseline diagnosis of AAA, were pregnant, or had missing data for inflammatory markers or other baseline information. After applying these exclusion criteria, the final analysis included 142,862 participants. A flowchart of the participant selection process is provided in Supplementary Figure S1.

### Dietary assessment and E-DII score calculation

Dietary intake was assessed using the web-based 24-h dietary recall tool, Oxford WebQ. Between April 2009 and June 2012, participants with valid email addresses were invited to complete the questionnaire up to five times (14). The WebQ collected frequency data on the consumption of 206 foods and 32 beverages over the previous 24 h. For each participant, an energy-adjusted Dietary Inflammatory Index (E-DII) score was calculated based on 30 dietary components, as listed in Supplementary Table S1.

Following the method proposed by Shivappa et al. (6), we computed E-DII scores through several steps. First, the average intake of each nutrient or food component was estimated. Second, energy adjustment was performed using the nutrient density method to control for total energy intake. Third, Z-scores were calculated by subtracting the global mean intake (from the reference database) from the individual’s energy-adjusted intake and dividing by the global standard deviation. These Z-scores were then converted to centered percentiles (percentile × 2–1). Finally, the centered percentile for each component was multiplied by its literature-derived inflammatory effect score, and all values were summed to yield the overall E-DII score. Higher E-DII values indicate a more pro-inflammatory diet. For categorical analyses, E-DII scores were grouped into tertiles: Tertile 1 (low), Tertile 2 (moderate), and Tertile 3 (high). In addition, standardized E-DII values (per 1-SD increase) were used as continuous variables in subsequent models.

### Polygenic risk score calculation

We created PRS using three separate approaches: a weighted PRS, PRS-CS (a Bayesian model with continuous shrinkage priors), and the clumping and thresholding (C + T) method. These methodologies have been thoroughly documented and compared in previous studies. First, we built the weighted PRS from 34 single nucleotide polymorphisms (SNPs) that were significantly associated with AAA, as shown in Supplementary Table S2. For each participant, we multiplied the number of risk alleles at each location by its effect size, and then summed these products to get the final score. Next, we used summary statistics from the largest GWAS of AAA in European ancestry populations, which we obtained from the GWAS Catalog, to implement the PRS-CS method. This approach estimates the posterior effect sizes of SNPs using a Bayesian regression model with continuous shrinkage priors, with the global shrinkage parameter set to its default value. For the C + T method, we performed linkage disequilibrium-based clumping. We used 1,000-kilobase windows, removed SNPs with high linkage disequilibrium (*r*^2^ > 0.1), and kept only the most significant SNP in each region. We then calculated the final PRS by summing the weighted effect sizes of the remaining variants.

To evaluate how well each PRS predicted outcomes, we calculated both the area under the receiver operating characteristic curve (AUC) and the concordance index (C-index). The PRS with the best predictive performance was chosen for all future analyses. We then categorized individuals into three genetic risk groups-low (bottom quintile), intermediate (2nd to 4th quintiles), and high (top quintile)-based on the distribution of this chosen PRS.

### Assessment of systemic inflammatory biomarkers

In the UK Biobank, all peripheral blood cell counts were measured using the clinically validated Coulter LH 750 automated hematology analyzer, with quality control procedures conducted in accordance with the manufacturer’s recommendations. We obtained baseline measurements of neutrophil, monocyte, platelet, and lymphocyte counts. Based on these values, two systemic inflammatory indices were calculated to reflect immune-inflammatory status: the SIRI, defined as neutrophil count multiplied by monocyte count divided by lymphocyte count, and the SII, defined as neutrophil count multiplied by platelet count divided by lymphocyte count. In addition, we assessed nutritional status using the PNI, calculated as serum albumin concentration plus five times the total lymphocyte count, and the CONUT score, which integrates serum albumin, total lymphocyte count, and total cholesterol levels to reflect overall nutritional and immune status.

### Covariate assessment

Covariates encompassed demographic characteristics (age, sex, and body mass index), socioeconomic indicators (employment status, Townsend deprivation index, and educational attainment), lifestyle variables, baseline clinical history (diabetes, hypertension, dyslipidemia, other cardiovascular diseases, chronic respiratory disease, chronic kidney disease, chronic liver disease, and cancer), and prior use of medications and vitamin supplements. Lifestyle variables included sleep pattern (healthy, intermediate, or unhealthy), physical activity level (high, moderate, or low), sedentary behavior (low, moderate, or high), and smoking history. Detailed definitions of each lifestyle component are provided in Supplementary Table S3.

### Outcomes

The primary outcome of this study was the occurrence of AAA, identified using ICD 10 codes I71.3 and I71.4. Incident AAA cases were ascertained through multiple data sources, including death registries, primary care records, hospital inpatient data, and self-reported medical diagnoses. The date of AAA onset was determined as the earliest recorded instance of a relevant diagnosis. Follow-up duration was measured from the date of the first completed 24-h dietary recall (collected between 2009 and 2012) to the earliest of the following events: AAA diagnosis, death, loss to follow-up, or the study’s end date.

### Statistical analysis

Baseline characteristics were described using standard descriptive methods. Means and standard deviations (SD) were used to summarize continuous variables, while categorical variables were reported as counts and proportions. Comparisons across groups were performed using one-way ANOVA for continuous variables and chi-square tests for categorical variables. Kaplan–Meier survival curves were generated to depict the cumulative incidence of AAA in relation to E-DII scores. Cox proportional hazards regression models were employed to assess the association between E-DII scores and incident AAA, with hazard ratios (HRs) and corresponding 95% confidence intervals (CIs) provided. The proportional hazards assumption was evaluated using Schoenfeld residuals. Three progressive multivariable models were developed: Model 1 adjusted for age, sex, education level, employment status, Townsend deprivation index, and body mass index (BMI); Model 2 included all variables in Model 1 plus adjustment for baseline comorbidities (hypertension, diabetes, dyslipidemia, cardiovascular disease, cancer, chronic respiratory disease, chronic kidney disease, and chronic liver disease), as well as prior use of medications and vitamin supplements; Model 3 further incorporated lifestyle factors, including smoking status, physical activity, sleep pattern, and sedentary behavior. To investigate potential non-linear relationships between E-DII and AAA risk, restricted cubic spline regression was applied. Stratified analyses were conducted to explore effect modification by age, sex, BMI category (normal: 18.5–24.9 kg/m^2^ vs. outside this range), history of hypertension, and smoking status.

A series of sensitivity analyses were conducted to evaluate the robustness and reliability of the results. First, to minimize potential reverse causation, cases diagnosed within the initial 2 years of follow-up were excluded. Second, participants with major baseline comorbidities (such as diabetes, hypertension, dyslipidemia, cardiovascular diseases, chronic liver or kidney disease, chronic respiratory conditions, and cancer) were removed to assess the influence of pre-existing health conditions. Third, to account for potential competing risks from non-AAA-related mortality or loss to follow-up, we applied a Fine-Gray sub-distribution hazards model. Fourth, multiple imputation techniques were used to address missing values in covariates and assess the influence of incomplete data. Finally, to examine the stability of the dietary exposure measure, the association between dietary inflammatory potential and AAA risk was reanalyzed among individuals who completed at least two 24-h dietary recalls.

To evaluate the potential influence of genetic predisposition, Cox proportional hazards models were initially employed to compare AAA risk across different strata of the PRS. We then investigated both the interaction and combined effects of E-DII scores and PRS on AAA incidence. Multiplicative interaction was assessed by incorporating a cross-product term between E-DII and PRS into the Cox model, with HRs and 95% CIs reported. Additive interaction was examined by calculating the relative excess risk due to interaction (RERI) and its corresponding 95% CI. For the joint effects analysis, individuals classified as having both low genetic risk and low E-DII scores served as the reference category. Finally, causal mediation analysis was conducted to evaluate the potential mediating role of systemic inflammatory indices (SIRI) in the association between the E-DII score and the risk of AAA, with adjustment for covariates included in Model 3. To estimate the 95% CIs for the proportion mediated, we performed 1,000 iterations of quasi-Bayesian Monte Carlo simulations using a bootstrap resampling approach. We used the maximally selected rank statistics combined with multiple testing correction to determine the optimal cut-off values for the E-DII score, PRS, and SIRI.

Statistical analyses were performed using R version 4.4.1 (R Foundation for Statistical Computing). All tests were two-tailed, and a *p*-value less than 0.05 was regarded as statistically significant.

## Results

### Baseline characteristics of participants

Baseline characteristics of the participants are presented in Table 1. A total of 142,862 individuals were included in the analysis, with a mean age of 56.2 years (SD = 7.9), and 45.9% were male. Compared to those in the lowest tertile of the E-DII score, participants in the highest tertile were more likely to be male, have lower educational attainment, exhibit less healthy lifestyle behaviors, report more comorbid conditions and medication use, and display higher levels of systemic immune-inflammation indices.

**Table 1:** Baseline characteristics of study participants according to E-DII score categories.

Characteristics	E-DII score			*p*-value
	Tertile 1	Tertile 2	Tertile 3	
*N*	47,621	47,620	47,621	
E-DII scores (range)	(−6.9, −1.4)	(−1.4, 1.2)	(1.2, 5.9)	
Demographics				
Age (years)	57.3 ± 7.52	56.3 ± 7.85	55.1 ± 8.09	<0.001
Male (%)	16,723 (35.1%)	22,196 (46.6%)	26,605 (55.9%)	<0.001
Townsend deprivation index	−1.9 ± 2.7	−1.8 ± 2.6	−1.6 ± 2.9	<0.001
University or college degree (%)	21,927 (46.0%)	21,999 (46.2%)	19,431 (40.8%)	<0.001
Employed, student, or retired (%)	44,145 (92.7%)	44,180 (92.8%)	43,894 (92.2%)	<0.001
BMI	26.5 ± 4.4	26.7 ± 4.3	27.3 ± 4.6	<0.001
Lifestyle				
Never smoking (%)	27,755 (58.3%)	27,331 (57.4%)	25,590 (53.7%)	<0.001
Physical activity (%)				<0.001
Low	11,061 (23.2%)	12,533 (26.3%)	15,050 (31.6%)	
Moderate	16,418 (34.5%)	16,511 (34.7%)	16,032 (33.7%)	
High	20,142 (42.3%)	18,576 (39.0%)	16,539 (34.7%)	
Sleep patterns (%)				<0.001
Poor	18,721 (39.3%)	17,297 (36.3%)	15,326 (32.2%)	
Moderate	27,185 (57.1%)	28,341 (59.5%)	29,683 (62.3%)	
Good	1715 (3.60%)	1982 (4.16%)	2,612 (5.48%)	
Sedentary time (%)				<0.001
High	9,132 (19.2%)	10,078 (21.2%)	11,870 (24.9%)	
Moderate	15,756 (33.1%)	15,794 (33.2%)	16,136 (33.9%)	
Low	22,733 (47.7%)	21,748 (45.7%)	19,615 (41.2%)	
Medical history				
Hypertension (%)	13,182 (27.7%)	13,086 (27.5%)	13,359 (28.1%)	0.135
Diabetes (%)	2,611 (5.48%)	2,659 (5.58%)	2,897 (6.08%)	<0.001
Dyslipidemia (%)	16,144 (33.9%)	17,688 (37.1%)	19,708 (41.4%)	<0.001
Cancer (%)	6,546 (13.7%)	6,040 (12.7%)	5,693 (12.0%)	<0.001
Chronic respiratory diseases (%)	6,071 (12.7%)	6,012 (12.6%)	6,403 (13.4%)	<0.001
Chronic liver disease (%)	99 (0.21%)	104 (0.22%)	143 (0.30%)	<0.001
Chronic kidney disease (%)	127 (0.27%)	139 (0.29%)	154 (0.32%)	0.271
Cardiovascular disease (%)	4,665 (9.80%)	4,520 (9.49%)	4,498 (9.45%)	0.136
Number of medications (SD)	2.2 ± 2.4	2.1 ± 2.3	2.0 ± 2.3	<0.001
Vitamin use (%)	14,938 (31.4%)	12,726 (26.7%)	8,392 (17.6%)	<0.001
Inflammation				
Neutrophil count (10^9^/L)	4.0 ± 1.2	4.1 ± 1.2	4.2 ± 1.2	<0.001
Monocyte count (10^9^/L)	0.4 ± 0.1	0.5 ± 0.2	0.5 ± 0.2	<0.001
Lymphocyte count (10^9^/L)	1.9 ± 0.5	1.9 ± 0.5	1.9 ± 0.5	<0.001
Platelet count (10^9^/L)	248 ± 51.8	248 ± 51.8	249 ± 51.9	<0.001
Albumin (g/L)	45.5 ± 2.5	45.5 ± 2.5	45.4 ± 2.6	0.006
Total cholesterol (mmol/L)	5.7 ± 1.1	5.7 ± 1.1	5.7 ± 1.1	<0.001
PNI	55.0 ± 3.7	54.9 ± 3.7	55.0 ± 3.8	0.352
CONUT	0.6 ± 0.7	0.6 ± 0.7	0.6 ± 0.7	0.984
SIRI	1.0 ± 0.5	1.1 ± 0.6	1.1 ± 0.6	<0.001
SII	563 ± 251	569 ± 255	580 ± 262	<0.001

*p-values were determined using the ANOVA test for continuous variables and the chi-square test for categorical variables. E-DII, energy-adjusted dietary inflammatory index; BMI, body mass index; SIRI, systemic inflammation response index; SII, systemic immune-inflammation index; PNI, prognostic nutritional index; CONUT, controlling nutritional status.*

### Association between the E-DII score and the risk of AAA

Over a mean follow-up of 13.8 years, a total of 483 incident AAA cases were documented. As shown in the Kaplan–Meier curves in Supplementary Figure S2, higher E-DII scores were significantly associated with increased AAA incidence. In multivariable Cox regression analyses (Table 2), participants in the highest tertile of the E-DII score had a significantly greater risk of AAA compared to those in the lowest tertile (HR = 1.36; 95% CI: 1.09–1.71). Additionally, each 1 SD increase in the E-DII score was associated with a 12% higher risk of AAA. Proportional hazards assumptions were verified using Schoenfeld residual tests, with all *p*-values > 0.05 across models (Supplementary Table S4), supporting the validity and robustness of the analyses. The C-index ranged from 0.846 to 0.871, with Model 3 showing the best model fit as indicated by the highest C-index and lowest Bayesian Information Criterion (BIC).

**Table 2:** Association between the E-DII score and AAA incidence.

Analysis	E-DII score HR (95%CI)				*p*-value
	Tertile 1	Tertile 2	Tertile 3	Per SD increment	
Cases/participants	124/47621	154/47620	205/47621	483/142862	
Model 1	1 (reference)	1.14 (0.90–1.44)	1.45 (1.16–1.82)	1.16 (1.05–1.27)	0.003
Model 2	1 (reference)	1.14 (0.90–1.45)	1.45 (1.16–1.82)	1.16 (1.05–1.27)	0.003
Model 3	1 (reference)	1.11 (0.88–1.41)	1.36 (1.09–1.71)	1.12 (1.02–1.23)	0.017

Model 1: Adjusted for sex, age, education level, employment status, Townsend deprivation index, and body mass index (BMI). Model 2: Further adjusted for prior medication use, vitamin supplementation, and medical history (including hypertension, diabetes, dyslipidemia, cardiovascular and cerebrovascular diseases, cancer, chronic respiratory diseases, chronic kidney disease, and chronic liver disease). Model 3: Additionally adjusted for smoking history, physical activity, sleep pattern, and sedentary time. E-DII, energy-adjusted diet inflammatory index; HR, hazard ratio; CI, confidence interval; SD, standard deviation.

Subgroup analyses (Supplementary Table S5) revealed no significant interactions between the E-DII score and AAA incidence across subgroups stratified by sex, age, BMI, hypertension status, or smoking history. Several sensitivity analyses were conducted to test the robustness of the findings. As shown in Supplementary Table S6, the positive association between the E-DII score and AAA risk remained significant after excluding cases occurring within the first 2 years of follow-up, excluding participants with preexisting diseases, applying a competing risk model, or restricting the analysis to participants who completed at least two 24-h dietary assessments. These findings further support the consistency and robustness of our results.

**Figure 1:**
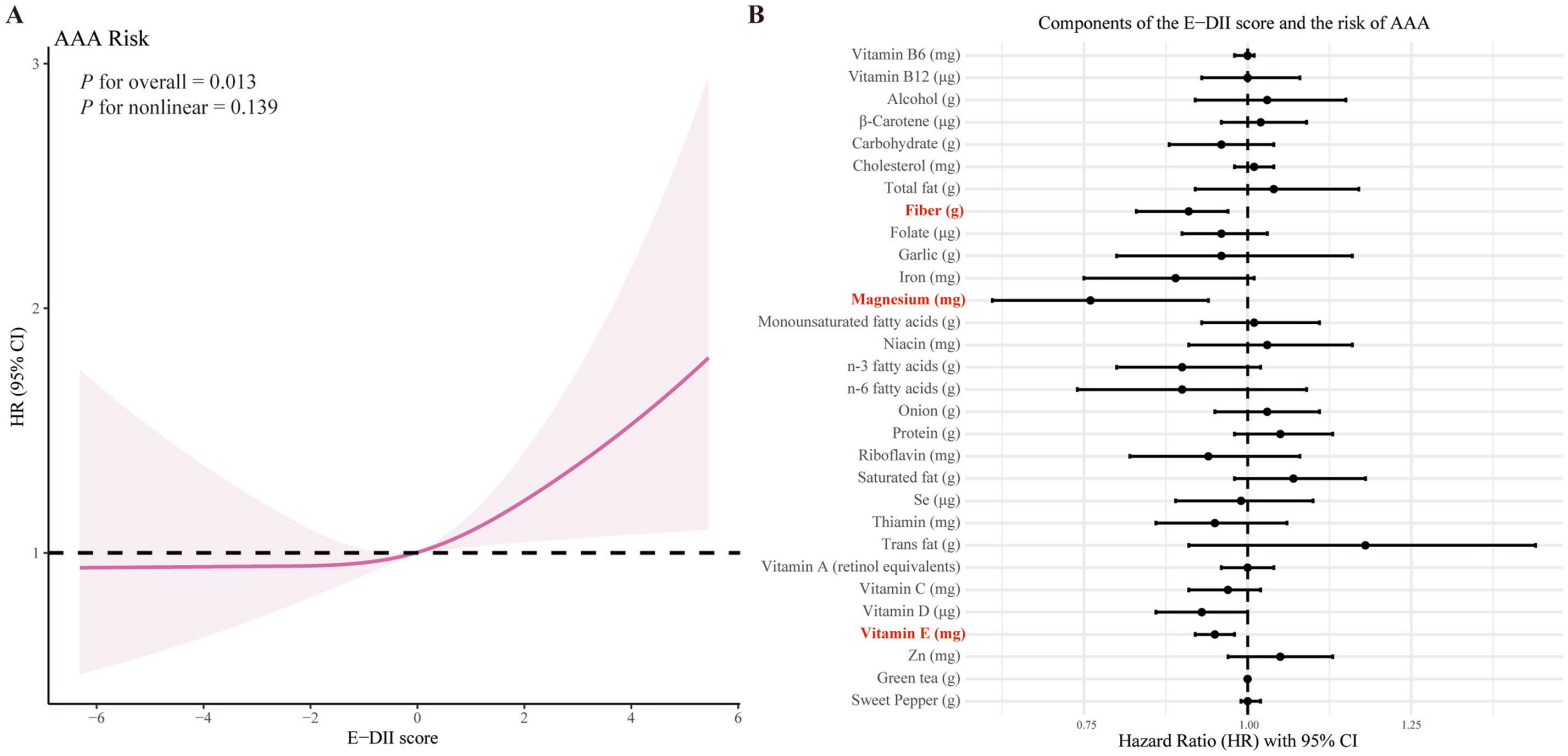
Association of the E-DII score and its components with AAA risk. **(A)** Restricted cubic spline analysis of the association between E-DII score and AAA risk. **(B)** Associations of individual E-DII components with the risk of AAA. Analyzes were adjusted for sex, age, education level, employment status, Townsend deprivation index, body mass index, prior medication use, vitamin supplementation, medical history (encompassing hypertension, diabetes, dyslipidemia, cardiovascular and cerebrovascular diseases, cancer, chronic respiratory diseases, chronic kidney disease, and chronic liver disease), smoking history, physical activity, sleep pattern, and sedentary time.

Restricted cubic spline analysis (Figure 1A) demonstrated a linear dose–response relationship between the E-DII score and AAA incidence. As shown in Supplementary Figure S3, we next applied the maximally selected rank statistics method with multiple testing adjustment to determine the optimal cut-off value for the E-DII. The final cut-off was identified as 1.073, which bootstrap resampling yielded a median HR of 1.44, indicating an overall consistent trend. Figure 1B illustrates the associations between individual E-DII components and AAA risk. After adjustment for all potential confounders, higher intakes of fiber, magnesium, and vitamin E were significantly associated with reduced AAA risk, while no significant associations were observed for other dietary components.

### Genetic risk and its interaction with the E-DII score in relation to AAA risk

We constructed PRS for AAA using three different methods. As shown in Supplementary Table S7 and Supplementary Figure S4, all three PRS approaches were significantly associated with AAA incidence. Among them, the PRS generated using the PRS-CS method demonstrated the highest predictive performance, as indicated by the highest C-index and AUC values. Therefore, subsequent analyses in our study were primarily based on the PRS-CS results. Based on the maximally selected rank statistics method, the cut-off value for PRS was determined to be −0.003. Compared to individuals in the lowest quintile of genetic risk, those in the highest quintile had a significantly increased risk of AAA (HR = 2.35; 95% CI: 2.02–3.32).

**Figure 2:**
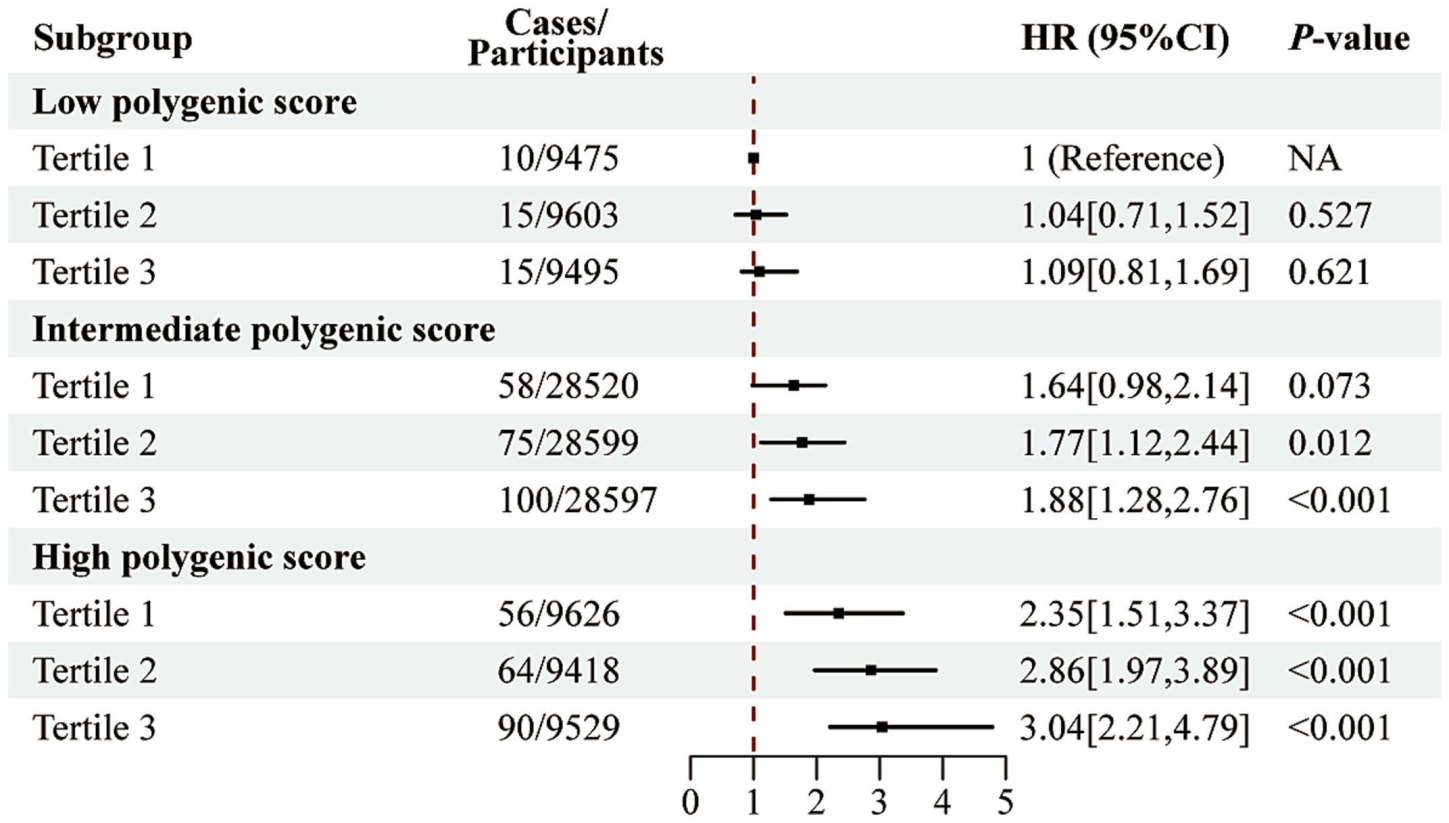
Joint association of the E-DII score and polygenic risk score with AAA risk. Analyzes were adjusted for sex, age, education level, employment status, Townsend deprivation index, body mass index, prior medication use, vitamin supplementation, medical history (encompassing hypertension, diabetes, dyslipidemia, cardiovascular and cerebrovascular diseases, cancer, chronic respiratory diseases, chronic kidney disease, and chronic liver disease), smoking history, physical activity, sleep pattern, and sedentary time.

As illustrated in Supplementary Figure S5, no significant interaction was observed between PRS and the E-DII score in relation to AAA risk. However, as shown in Figure 2, the combined effect of PRS and E-DII score revealed a markedly increased risk. Specifically, individuals with both high PRS and high E-DII scores had approximately a 3.04-fold higher risk of developing AAA compared to those with low PRS and low E-DII scores (HR = 3.04; 95% CI: 2.21–4.79).

### Associations of inflammatory indices and the E-DII score with AAA risk

As shown in Supplementary Table S8, after adjusting for all potential confounders, higher levels of the SIRI were significantly associated with an increased risk of AAA (HR = 1.57; 95% CI: 1.14–2.16). Additionally, each 1-standard deviation increase in SIRI was associated with a 10% higher risk of AAA (HR = 1.10; 95% CI: 1.01–1.20). In contrast, no significant associations were observed between SII, PNI, CONUT, and the risk of AAA. Subsequently, using the maximally selected rank statistics method, the cut-off value for SIRI was determined to be 1.22.

**Figure 3:**
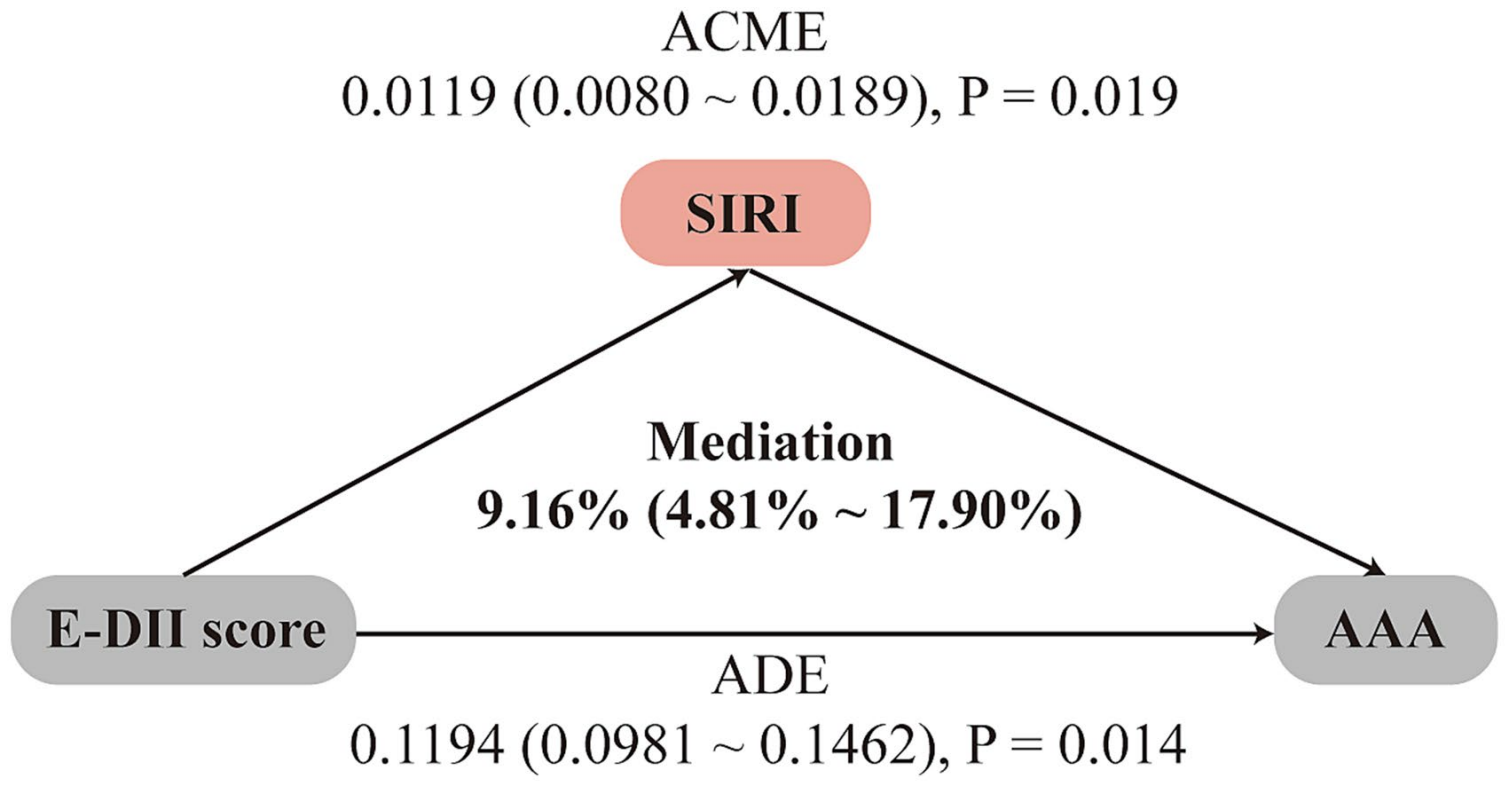
Mediation analysis of inflammatory index SIRI in the association between E-DII score and AAA risk. Analyzes were adjusted for sex, age, education level, employment status, Townsend deprivation index, body mass index, prior medication use, vitamin supplementation, medical history (encompassing hypertension, diabetes, dyslipidemia, cardiovascular and cerebrovascular diseases, cancer, chronic respiratory diseases, chronic kidney disease, and chronic liver disease), smoking history, physical activity, sleep pattern, and sedentary time. ADE, average direct effects; ACME, average causal mediation effects.

We further investigated the potential mediating role of SIRI in the association between the E-DII score and the risk of AAA. As shown in Figure 3, SIRI was found to mediate 9.16% of the association between E-DII and AAA risk (95% CI: 4.81%–17.90%).

## Discussion

In this study, we found that higher E-DII scores were significantly associated with an increased risk of AAA. Moreover, the association between E-DII and AAA risk appeared to follow an upward trend across individuals with low, moderate, and high levels of PRS. Although no statistically significant interaction was detected, individuals with both high PRS and high E-DII scores exhibited the greatest risk of developing AAA. Notably, part of the association between E-DII and AAA risk was mediated by elevated levels of the SIRI.

In recent years, healthy dietary patterns have gained increasing attention as feasible and non-invasive strategies for disease prevention, particularly compared to surgical or pharmacological interventions. However, compared with other cardiovascular diseases, studies investigating the relationship between dietary patterns and the risk of AAA remain limited. Joanna et al. (15) developed an Anti-Inflammatory Diet Index (AIDI) based on 16 food items using data from two large Swedish cohorts comprising approximately 80,000 participants, and reported that adherence to an anti-inflammatory diet was associated with a reduced risk of AAA. These findings are consistent with our results. However, our analysis is based on a larger baseline population and incorporates a more comprehensive set of potential confounders.

The E-DII score reflects chronic low-grade systemic inflammation driven by dietary intake and supports the hypothesis that inflammation plays a key role in AAA pathogenesis, as suggested by previous evidence (16). Specifically, we observed that higher intakes of fiber, magnesium, and vitamin E were associated with a lower risk of AAA, compared with other nutrients. Dietary fiber may exert its protective effect through fermentation by the gut microbiota, producing short-chain fatty acids that modulate immune responses and reduce chronic inflammation in the vascular wall (17). Supporting this, Sara et al. (18) reported an independent association between high fiber intake and reduced AAA risk in a cohort of over 20,000 individuals. Both magnesium and vitamin E possess well-documented anti-inflammatory and antioxidant properties (19, 20). However, their roles in AAA development have been less thoroughly studied. In one animal study, Gavrila et al. (21) found that vitamin E supplementation significantly reduced maximal aortic diameter and rupture events in a mouse model of AAA. Taken together, these findings underscore the need for well-designed randomized controlled trials to further elucidate the potential dose-dependent effects of fiber, magnesium, and vitamin E on AAA development.

We also investigated the potential role of systemic inflammatory biomarkers, specifically SII and SIRI, in relation to AAA risk. To date, prospective cohort studies examining these indices in the context of AAA are scarce; most existing evidence is derived from retrospective studies, which have suggested that both SII and SIRI may serve as independent predictors of adverse outcomes following AAA surgery (22, 23). In our analysis, we found that elevated SIRI was significantly associated with increased AAA risk, whereas no such association was observed for SII. Furthermore, SIRI was shown to mediate approximately 9.16% of the association between the E-DII score and AAA risk, highlighting its potential role in the inflammatory pathway linking diet to AAA development. The differential associations observed between SII and SIRI may reflect the distinct biological components and inflammatory pathways captured by each index. In the case of SII, the inclusion of platelet count introduces complexity, as the role of platelets in AAA pathogenesis appears to be stage-dependent (24). Some studies have suggested that in the early phase of AAA development, platelets may exert protective effects by modulating initial inflammation and stabilizing intraluminal thrombus (25). However, in later stages, platelet activation may promote leukocyte infiltration and amplify vascular inflammation, thereby accelerating aneurysm expansion (26). This dual role could partly explain the absence of a significant association between SII and AAA risk in our prospective analysis. By contrast, SIRI incorporates neutrophil, monocyte, and lymphocyte counts, components that may more accurately reflect the chronic inflammatory milieu involved in AAA progression (27). In addition, neither PNI nor CONUT scores showed significant associations with AAA risk. These results indicate that not all nutrition-related indices are equally informative in predicting AAA occurrence. As such, SIRI may be a more sensitive marker of inflammation-driven AAA risk. Nonetheless, further prospective cohort studies are needed to confirm these findings and to elucidate the mechanistic pathways underlying the observed associations.

In recent years, GWAS based PRS have shown strong utility in quantifying genetic susceptibility to various cardiovascular diseases. For example, Kelemen et al. (28) developed a PRS using data from multiple biobank cohorts and demonstrated that higher PRS was significantly associated with increased AAA risk, findings that are consistent with our results. Notably, our study further elucidates, for the first time, the complex interplay between genetic predisposition and dietary inflammation in the development of AAA. Although no statistically significant interaction was observed between PRS and the E-DII score, joint analyses revealed that individuals with both high genetic risk and high dietary inflammatory potential exhibited a markedly increased risk of AAA. This finding underscores a potential additive effect of genetic susceptibility and lifestyle-related factors. It also aligns with prior research in other cardiovascular conditions, where individuals at elevated genetic risk were shown to benefit from healthy lifestyle modifications (29). These findings suggest that personalized dietary recommendations based on genetic background may have a role in the prevention of AAA. However, further large-scale prospective studies and mechanistic investigations are warranted to better understand the relationship between genetic susceptibility and pro-inflammatory dietary patterns in the context of AAA.

This study has several notable strengths. First, it is based on a large-scale prospective cohort design, which enhances the robustness and reliability of the findings. Second, to our knowledge, this is the first study to systematically evaluate the association between an inflammatory dietary score and the risk of AAA. In addition, we assessed the potential roles of genetic susceptibility and dietary patterns in AAA development, and further explored whether systemic inflammatory indices may mediate these associations. These findings offer novel insights and valuable guidance for future preventive strategies. However, several limitations should be acknowledged. First, the UK Biobank lacks detailed information on aneurysm diameter, preventing us from examining aneurysm size as an outcome. Second, although we adjusted for a wide range of potential confounders, the possibility of residual confounding (e.g., unmeasured comorbidities) cannot be entirely ruled out. Third, dietary intake was assessed using self-reported 24-h recall questionnaires, which may not fully capture long-term dietary exposures, especially given that dietary patterns can change over time. Lastly, the study population was limited to White British participants who completed the dietary assessments, which may restrict the generalizability of our findings to other racial and ethnic groups.

## Conclusion

In summary, our findings demonstrate that both the pro-inflammatory potential of diet and PRS are significantly associated with an increased risk of AAA. Individuals with higher genetic susceptibility may be more vulnerable to the detrimental effects of a pro-inflammatory diet. Moreover, the systemic inflammation response index may serve as a key mediator in the relationship between dietary inflammation and AAA development. These results underscore the potential value of integrating dietary patterns, genetic risk, and inflammatory biomarkers into targeted prevention strategies for AAA.

## Data Availability

The original contributions presented in the study are included in the article/Supplementary material, further inquiries can be directed to the corresponding author.
