# Supplementary material for: Dietary inflammatory potential, genetic susceptibility, and systemic inflammation indices in relation to abdominal aortic aneurysm risk: a prospective cohort study
Source: Front Nutr. 2025 Oct 17;12:1680225. doi: 10.3389/fnut.2025.1680225 (PMC12576294; doi:10.3389/fnut.2025.1680225)
Supplement: Supplementary file 1 [file Table_1.docx]

**Supplementary Material**

**Supplementary method**

**Supplementary Table S1.** Components and Scoring Details of the Energy-Adjusted Dietary Inflammatory Index (E-DII)

**Supplementary Table S2.** Information of genetic variants associated with AAA in the UK Biobank

**Supplementary Table S3.** Lifestyle assessment

**Supplementary Table S4.** Comparison of the predictive performance of diet quality scores in different Cox models

**Supplementary Table S5.** Subgroup associations between E-DII scores and AAA

**Supplementary Table S6.** Sensitivity analysis of the association between E-DII scores and the incidence of AAA

**Supplementary Table S7.** Association Between Polygenic Risk Score and the Incidence of AAA

**Supplementary Table S8.** Association between inflammatory index and the risk of AAA

**Supplementary Figure S1.** Flowchart of study participants

**Supplementary Figure S2.** Kaplan–Meier curves for cumulative incidence of AAA according to E-DII score categories

**Supplementary Figure S3.** Density plot of the E-DII and identified threshold

**Supplementary Figure S4.** Receiver Operating Characteristic Curves for Three Polygenic Risk Scores

**Supplementary Figure S5.** Interaction between the E-DII score and polygenic risk score in relation to AAA risk

**Supplementary Table S1.** Components and Scoring Details of the Energy-Adjusted Dietary Inflammatory Index (E-DII)

| **Components** | **Overall inflammatory effect score** | **Global daily mean intake (units/d)** | **SD** |
| --- | --- | --- | --- |
| Alcohol (g) | –0.278 | 13.98 | 3.72 |
| Vitamin B12 (μg) | 0.106 | 5.15 | 2.7 |
| Vitamin B6 (mg) | –0.365 | 1.47 | 0.74 |
| β-Carotene (μg) | –0.584 | 3718 | 1720 |
| Carbohydrate (g) | 0.097 | 272.2 | 40 |
| Cholesterol (mg) | 0.11 | 279·4 | 51.2 |
| Energy (kcal) | 0.18 | 2056 | 338 |
| Total fat (g) | 0.298 | 71.4 | 19.4 |
| Fiber (g) | –0.663 | 18.8 | 4.9 |
| Folate (μg) | –0.190 | 273 | 70.7 |
| Garlic (g) | –0.412 | 4.35 | 2.9 |
| Iron (mg) | 0.032 | 13.35 | 3.71 |
| Magnesium (mg) | –0.484 | 310.1 | 139.4 |
| Monounsaturated fatty acids (g) | –0.009 | 27 | 6.1 |
| Niacin (mg) | –0.246 | 25.9 | 11.77 |
| n-3 Fatty acids (g) | –0.436 | 1.06 | 1.06 |
| n-6 Fatty acids (g) | –0.159 | 10.8 | 7.5 |
| Onion (g) | –0.301 | 35.9 | 18.4 |
| Protein (g) | 0.021 | 79.4 | 13.9 |
| Riboflavin (mg) | –0.068 | 1.7 | 0.79 |
| Saturated fat (g) | 0.373 | 28.6 | 8 |
| Se (μg) | –0.191 | 67 | 25.1 |
| Thiamin (mg) | –0.098 | 1.7 | 0.66 |
| Trans fat (g) | 0.229 | 3.15 | 3.75 |
| Vitamin A (retinol equivalents) | –0.401 | 983.9 | 518.6 |
| Vitamin C (mg) | –0.424 | 118.2 | 43.46 |
| Vitamin D (μg) | –0.446 | 6.26 | 2.21 |
| Vitamin E (mg) | –0.419 | 8.73 | 1.49 |
| Zn (mg) | –0.313 | 9.84 | 2.19 |
| Green tea (g) | –0.536 | 1.69 | 1.53 |
| Sweet Pepper (g) | –0.131 | 10 | 7.07 |

**Supplementary Table S2.** Information of genetic variants associated with AAA in the UK Biobank

| **SNP** | **Gene** | **Effect allele** | **Other allele** | **Beta** | ***P-*value** | **Effect allele frequency** |
| --- | --- | --- | --- | --- | --- | --- |
| rs6658835 | TGFB2 | G | A | 0.1375 | 1.10E-08 | 0.2968 |
| rs76418310 |  | A | G | 2.8167 | 1.86E-06 | 0.0019 |
| rs28726382 |  | G | T | -0.2126 | 2.03E-06 | 0.0757 |
| rs11928580 | ULK4 | T | C | 0.1478 | 7.84E-07 | 0.1556 |
| rs77097530 |  | G | C | 0.2469 | 2.22E-06 | 0.0676 |
| rs56283118 |  | T | C | 0.7707 | 4.04E-06 | 0.0143 |
| rs4557486 | LINC01611 | T | C | -0.1676 | 2.33E-06 | 0.1559 |
| rs150678222 | EPHA7 | T | C | 0.555 | 4.32E-06 | 0.0139 |
| rs10250287 | SUGCT | C | T | -0.1078 | 1.11E-06 | 0.5615 |
| rs146303207 |  | A | G | 0.6108 | 4.19E-07 | 0.0113 |
| rs4977575 |  | G | C | 0.1301 | 6.03E-09 | 0.5239 |
| rs10818579 | DAB2IP | A | G | 0.1164 | 4.78E-06 | 0.249 |
| rs2689691 | PLCE1 | T | C | 0.1063 | 2.12E-06 | 0.4657 |
| rs662799 | APOA5 | A | G | -0.1678 | 5.49E-07 | 0.8584 |
| rs143484390 | LRRC4C | T | C | 0.5498 | 2.86E-06 | 0.0102 |
| rs2051466 | LINC02732 | A | C | -0.1138 | 3.05E-06 | 0.2876 |
| rs10894192 | ZBTB44-DT | A | T | 0.1362 | 2.45E-08 | 0.6593 |
| rs12824962 | LOC107984543 | A | G | 0.162 | 1.73E-06 | 0.1168 |
| rs11172113 | LRP1 | C | T | -0.1307 | 2.75E-08 | 0.3494 |
| rs9522384 |  | C | T | 0.1131 | 4.16E-06 | 0.7347 |
| rs9510086 |  | C | G | -0.206 | 3.00E-16 | 0.2974 |
| rs77291804 | G2E3-AS1 | A | T | 0.5685 | 4.97E-06 | 0.0129 |
| rs1992314 | LOXL1 | G | C | 0.1175 | 2.89E-07 | 0.3878 |
| rs58365910 |  | C | T | 0.1384 | 2.29E-07 | 0.2512 |
| rs2003956 | PLA2G4D | G | A | -0.1649 | 3.55E-06 | 0.4107 |
| rs1036476 | FBN1 | C | T | 0.2888 | 8.74E-10 | 0.0878 |
| rs79599475 | CDH13 | T | G | 0.4158 | 1.76E-06 | 0.0222 |
| rs2333625 | TUBD1 | C | T | -0.1301 | 4.79E-07 | 0.4343 |
| rs113193821 | CLUL1 | A | G | -0.33 | 4.94E-06 | 0.065 |
| rs7239677 |  | T | C | -0.1121 | 3.58E-06 | 0.7027 |
| rs112009052 | LTBP4 | A | T | -0.4661 | 3.86E-06 | 0.0158 |
| rs185863687 | DOT1L | T | C | -0.4333 | 1.75E-06 | 0.0165 |
| rs113096019 | MEIOSIN | G | A | 0.7587 | 2.88E-06 | 0.0103 |
| rs7270354 |  | A | G | 0.1643 | 6.48E-07 | 0.1297 |

**Supplementary Table S3.** Lifestyle assessment

| **Lifestyle factor** | **Field IDs** | **Lifestyle assessment** |
| --- | --- | --- |
| Smoking | 1239 1249 2644 | Past or current smoking  Smoked at least 100 cigarettes in their lifetime |
| Sleep pattern | 1160  1180 1200 1210 1220 | For each component of the sleep pattern, a score of 1 is assigned to high-risk components, while low-risk components are assigned a score of 0. The total sleep pattern score ranges from 0 to 5, with scores of 0–1 indicating a low-risk category, 2–3 representing a medium-risk category, and 4–5 corresponding to high-risk sleep patterns. |
| Physical activity | 971 981 991 1001 2624 2634 3637 3647 | The cohort was categorized into light, moderate, and heavy physical activity levels based on the tertile distribution of weekly physical activity Metabolic Equivalent of Task (MET) values. |
| Sedentary time | 1070  1080 | The cohort was divided into mild, moderate, and severe levels of sedentary behavior based on the tertile distribution of sedentary time. |

**Supplementary Table S4.** Comparison of the predictive performance of diet quality scores in different Cox models

| **Analysis** | **E-DII score** | |
| --- | --- | --- |
|  | **Categorical variable with three levels** | **Per SD increment** |
| **Model 1** |  |  |
| Schoenfeld Test (P-value) | 0.072 | 0.073 |
| C-index | 0.846 | 0.846 |
| BIC | 10641.54 | 10637.54 |
| **Model 2** |  |  |
| Schoenfeld Test (P-value) | 0.278 | 0.296 |
| C-index | 0.861 | 0.861 |
| BIC | 10598.64 | 10594.53 |
| **Model 3** |  |  |
| Schoenfeld Test (P-value) | 0.104 | 0.109 |
| C-index | 0.871 | 0.870 |
| BIC | 10543.74 | 10539.66 |

Model 1: Adjusted for sex, age, education level, employment status, Townsend Deprivation Index, and body mass index (BMI). Model 2: Further adjusted for prior medication use, vitamin supplementation, and medical history (including hypertension, diabetes, dyslipidemia, cardiovascular and cerebrovascular diseases, cancer, chronic respiratory diseases, chronic kidney disease, and chronic liver disease). Model 3: Additionally adjusted for smoking history, physical activity, sleep pattern, and sedentary time.

Abbreviations: BIC, Bayesian Information Criterion; E-DII, Energy‑adjusted Diet Inflammatory Index

**Supplementary Table S5.** Subgroup associations between E-DII scores and AAA

| **Subgroup** | **Cases/participants** | **Tertile 3 vs Tertile 1** | **Per SD increment** | ***P*_interaction_** |
| --- | --- | --- | --- | --- |
| **Sex** |  |  |  | 0.214 |
| Male | 415/65524 | 1.26 (0.99-1.61) | 1.09 (0.99-1.22) |  |
| Female | 68/77338 | 2.06 (1.11-2.81) | 1.23 (0.96-1.56) |  |
| **Age** |  |  |  | 0.782 |
| ≥60 | 387/59057 | 1.32 (1.03-1.70) | 1.12 (1.01-1.24) |  |
| <59 | 96/83805 | 1.66 (0.94-2.93) | 1.16 (0.94-1.45) |  |
| **BMI** |  |  |  | 0.218 |
| Normal BMI | 61/37591 | 2.14 (1.08-3.79) | 1.46 (1.10-1.94) |  |
| Abnormal BMI | 422/105271 | 1.26 (0.99-1.61) | 1.07 (0.97-1.19) |  |
| **Hypertension** |  |  |  | 0.339 |
| No | 226/103235 | 1.45 (1.04-2.03) | 1.16 (1.01-1.34) |  |
| Yes | 257/39627 | 1.28 (0.94-1.76) | 1.09 (0.96-1.24) |  |
| **Smoke** |  |  |  | 0.306 |
| No | 113/80676 | 1.03 (0.69-1.64) | 1.04 (0.86-1.26) |  |
| Yes | 370/62186 | 1.47 (1.13-1.92) | 1.14 (1.03-1.28) |  |

Analyzes were adjusted for sex, age, education level, employment status, Townsend Deprivation Index, body mass index, prior medication use, vitamin supplementation, medical history (encompassing hypertension, diabetes, dyslipidemia, cardiovascular and cerebrovascular diseases, cancer, chronic respiratory diseases, chronic kidney disease, and chronic liver disease), smoking history, physical activity, sleep pattern, and sedentary time.

**Supplementary Table S6.** Sensitivity analysis of the association between E-DII scores and the incidence of AAA

| **Analyses** | **E-DII score** | | **Schoenfeld Test (P value)** |
| --- | --- | --- | --- |
|  | **HR (95% CI)** | ***P*-value** |  |
| **Excluding events that occurred within the first two years of follow-up** | | |  |
| Tertile 3 vs Tertile 1 | 1.39 (1.11-1.74) | 0.002 | 0.265 |
| Per SD increment | 1.14 (1.03-1.26) | 0.003 | 0.214 |
| **Excludes individuals with hypertension, diabetes, cancer, dyslipidemia, CVD, CLD, CKD, or CRD** | | | |
| Tertile 3 vs Tertile 1 | 1.44 (1.01-1.84) | 0.032 | 0.289 |
| Per SD increment | 1.31 (1.02-1.72) | 0.036 | 0.275 |
| **Competing risk regression model** | | |  |
| Tertile 3 vs Tertile 1 | 1.30 (1.05-1.61) | 0.011 | NA |
| Per SD increment | 1.11 (1.02-1.21) | 0.014 | NA |
| **Multiple imputation** | | |  |
| Tertile 3 vs Tertile 1 | 1.30 (1.05-1.59) | 0.010 | 0.215 |
| Per SD increment | 1.11 (1.03-1.22) | 0.007 | 0.224 |
| **Exclude participants who only completed one dietary assessment** | | |  |
| Tertile 3 vs Tertile 1 | 1.35 (1.03-1.80) | 0.021 | 0.578 |
| Per SD increment | 1.21 (1.07-1.37) | 0.002 | 0.465 |

Analyzes were adjusted for sex, age, education level, employment status, Townsend Deprivation Index, body mass index, prior medication use, vitamin supplementation, medical history (encompassing hypertension, diabetes, dyslipidemia, cardiovascular and cerebrovascular diseases, cancer, chronic respiratory diseases, chronic kidney disease, and chronic liver disease), smoking history, physical activity, sleep pattern, and sedentary time.

**Supplementary Table S7.** Association Between Polygenic Risk Score and the Incidence of AAA

| **Genetic risk** | **Cases/**  **participants** | **Model 1** | | **Model 2** | | **Model 3** | | |
| --- | --- | --- | --- | --- | --- | --- | --- | --- |
|  |  | **HR (95% CI)** | **C-index** | **HR (95% CI)** | **C-index** | **HR (95% CI)** | ***P*-value** | **C-index** |
| **PRS-CS** |  |  | 0.864 |  | 0.875 |  |  | 0.883 |
| Low (bottom quintile) | 40/28573 | 1 (Reference) |  | 1 (Reference) |  | 1 (Reference) | NA |  |
| Intermediate (quintiles 2-4) | 233/85716 | 1.31 (1.03-1.54) |  | 1.30 (1.01-1.51) |  | 1.27 (1.01-1.46) | <0.001 |  |
| High (top quintile) | 210/28573 | 2.44 (2.09-3.45) |  | 2.39 (2.05-3.41) |  | 2.35 (2.02-3.32) | <0.001 |  |
| *P* for trend |  |  |  |  |  |  | <0.001 |  |
| **PRS(C+T)** |  |  | 0.860 |  | 0.869 |  |  | 0.878 |
| Low (bottom quintile) | 44/28525 | 1 (Reference) |  | 1 (Reference) |  | 1 (Reference) | NA |  |
| Intermediate (quintiles 2-4) | 235/85944 | 1.27 (1.04-1.48) |  | 1.24 (1.02-1.45) |  | 1.18 (1.01-1.41) | <0.001 |  |
| High (top quintile) | 204/28393 | 2.30 (1.95-3.09) |  | 2.25 (1.87-3.02) |  | 2.17 (1.70-2.95) | <0.001 |  |
| *P* for trend |  |  |  |  |  |  | <0.001 |  |
| **Weighted PRS** | |  | 0.848 |  | 0.861 |  |  | 0.871 |
| Low (bottom quintile) | 37/28604 | 1 (Reference) |  | 1 (Reference) |  | 1 (Reference) | NA |  |
| Intermediate (quintiles 2-4) | 265/85735 | 1.31 (1.05-1.60) |  | 1.28 (1.06-1.55) |  | 1.29 (1.04-1.58) | <0.001 |  |
| High (top quintile) | 181/28523 | 2.18 (1.84-2.71) |  | 2.10 (1.71-2.81) |  | 2.09 (1.67-2.75) | <0.001 |  |
| *P* for trend |  |  |  |  |  |  | <0.001 |  |

Model 1: Adjusted for sex, age, education level, employment status, Townsend Deprivation Index, and body mass index. Model 2: Further adjusted for prior medication use, vitamin supplementation, and medical history (including hypertension, diabetes, dyslipidemia, cardiovascular and cerebrovascular diseases, cancer, chronic respiratory diseases, chronic kidney disease, and chronic liver disease). Model 3: Additionally adjusted for smoking history, physical activity, sleep pattern, and sedentary time. Abbreviations: PRS, Polygenic risk score; CI, confidence interval; HR, hazard ratio.

**Supplementary Table S8.** Association between inflammatory index and the risk of AAA

| **Inflammatory index** | **Cases/**  **participants** | **Model 1** | **Model 2** | **Model 3** |
| --- | --- | --- | --- | --- |
| **SII** |  |  |  |  |
| Quartile 1 | 117/35716 | 1 (Reference) | 1 (Reference) | 1 (Reference) |
| Quartile 2 | 115/35715 | 1.02 (0.79-1.32) | 1.01 (0.77-1.30) | 0.99 (0.76-1.28) |
| Quartile 3 | 132/35715 | 1.17 (0.91-1.50) | 1.13 (0.88-1.45) | 1.12 (0.87-1.43) |
| Quartile 4 | 119/35716 | 1.03 (0.80-1.33) | 0.97 (0.75-1.26) | 0.96 (0.74-1.24) |
| *P* for trend |  | 0.486 | 0.838 | 0.936 |
| Per SD increment | 483/142862 | 1.03 (0.94-1.13) | 1.01 (0.92-1.10) | 1.02 (0.92-1.12) |
| **SIRI** |  |  |  |  |
| Quartile 1 | 49/35716 | 1 (Reference) | 1 (Reference) | 1 (Reference) |
| Quartile 2 | 99/35715 | 1.47 (1.04-2.07) | 1.42 (1.01-2.01) | 1.42 (1.01-2.01) |
| Quartile 3 | 129/35715 | 1.55 (1.12-2.17) | 1.45 (1.04-2.03) | 1.45 (1.04-2.02) |
| Quartile 4 | 206/35716 | 1.81 (1.31-2.49) | 1.61 (1.17-2.22) | 1.57 (1.14-2.16) |
| *P* for trend |  | 0.001 | 0.018 | 0.032 |
| Per SD increment | 483/142862 | 1.16 (1.07-1.26) | 1.11 (1.02-1.21) | 1.10 (1.01-1.20) |
| **PNI** |  |  |  |  |
| Quartile 1 | 136/35716 | 1 (Reference) | 1 (Reference) | 1 (Reference) |
| Quartile 2 | 125/35716 | 1.05 (0.82-1.34) | 1.04 (0.82-1.33) | 1.04 (0.81-1.32) |
| Quartile 3 | 110/35715 | 0.99 (0.77-1.28) | 0.98 (0.76-1.26) | 0.96 (0.74-1.23) |
| Quartile 4 | 112/35715 | 1.12 (0.87-1.45) | 1.07 (0.83-1.38) | 1.02 (0.79-1.32) |
| *P* for trend |  | 0.793 | 0.888 | 0.626 |
| Per SD increment | 483/142862 | 1.01 (0.98-1.03) | 1.01 (0.97-1.02) | 0.99 (0.97-1.02) |
| **CONUT** |  |  |  |  |
| Normal nutrition (Score 0-1) | 411/125570 | 1 (Reference) | 1 (Reference) | 1 (Reference) |
| Malnutrition (Score ≥2) | 72/17292 | 0.83 (0.65-1.07) | 0.88 (0.72-1.13) | 0.91 (0.73-1.15) |

Model 1: Adjusted for sex, age, education level, employment status, Townsend Deprivation Index, and body mass index. Model 2: Further adjusted for prior medication use, vitamin supplementation, and medical history (including hypertension, diabetes, dyslipidemia, cardiovascular and cerebrovascular diseases, cancer, chronic respiratory diseases, chronic kidney disease, and chronic liver disease). Model 3: Additionally adjusted for smoking history, physical activity, sleep pattern, and sedentary time. Abbreviations: SII, Systemic Immune-Inflammation Index; SIRI, Systemic Inflammation Response Index; PNI, Prognostic Nutritional Index; CONUT, Controlling Nutritional Status.

**Supplementary Figure S1.** Flowchart of study participants


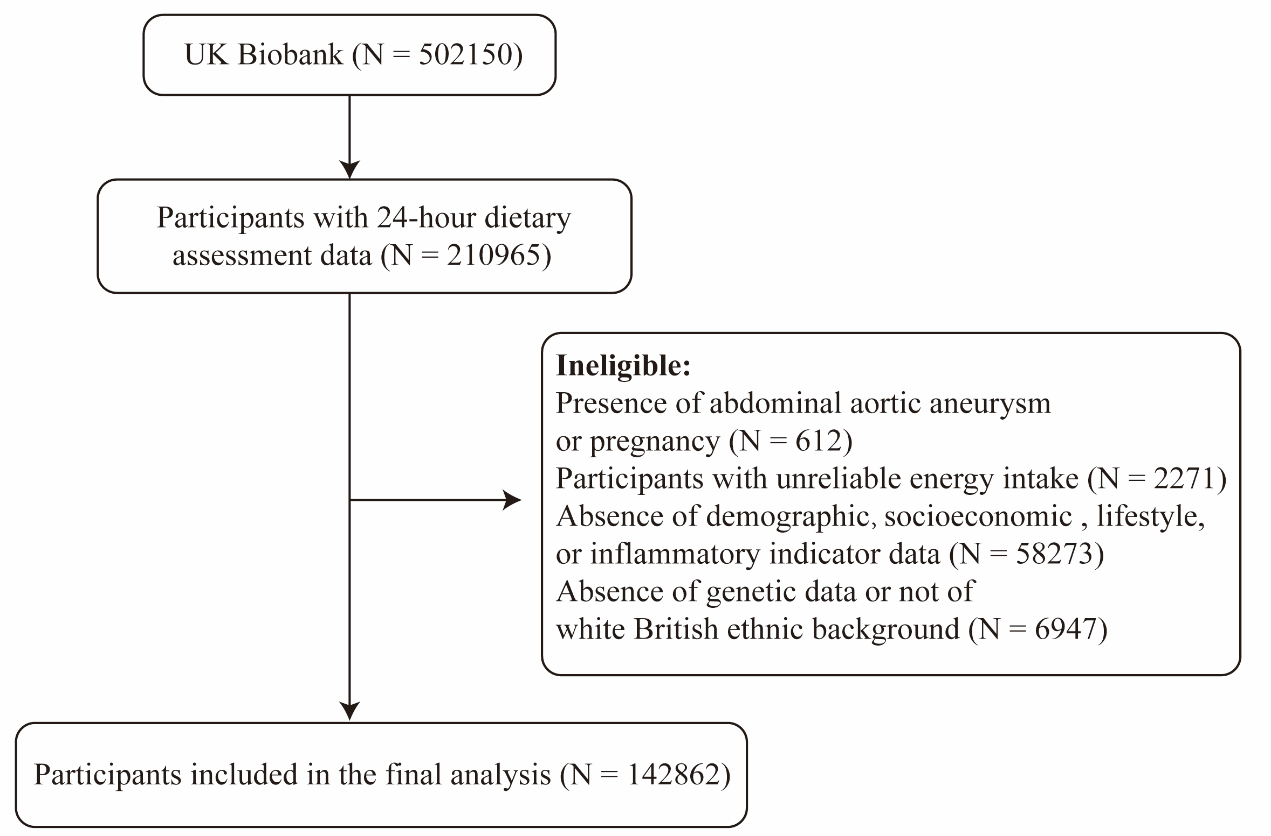


**Supplementary Figure S2.** Kaplan–Meier curves for cumulative incidence of AAA according to E-DII score categories


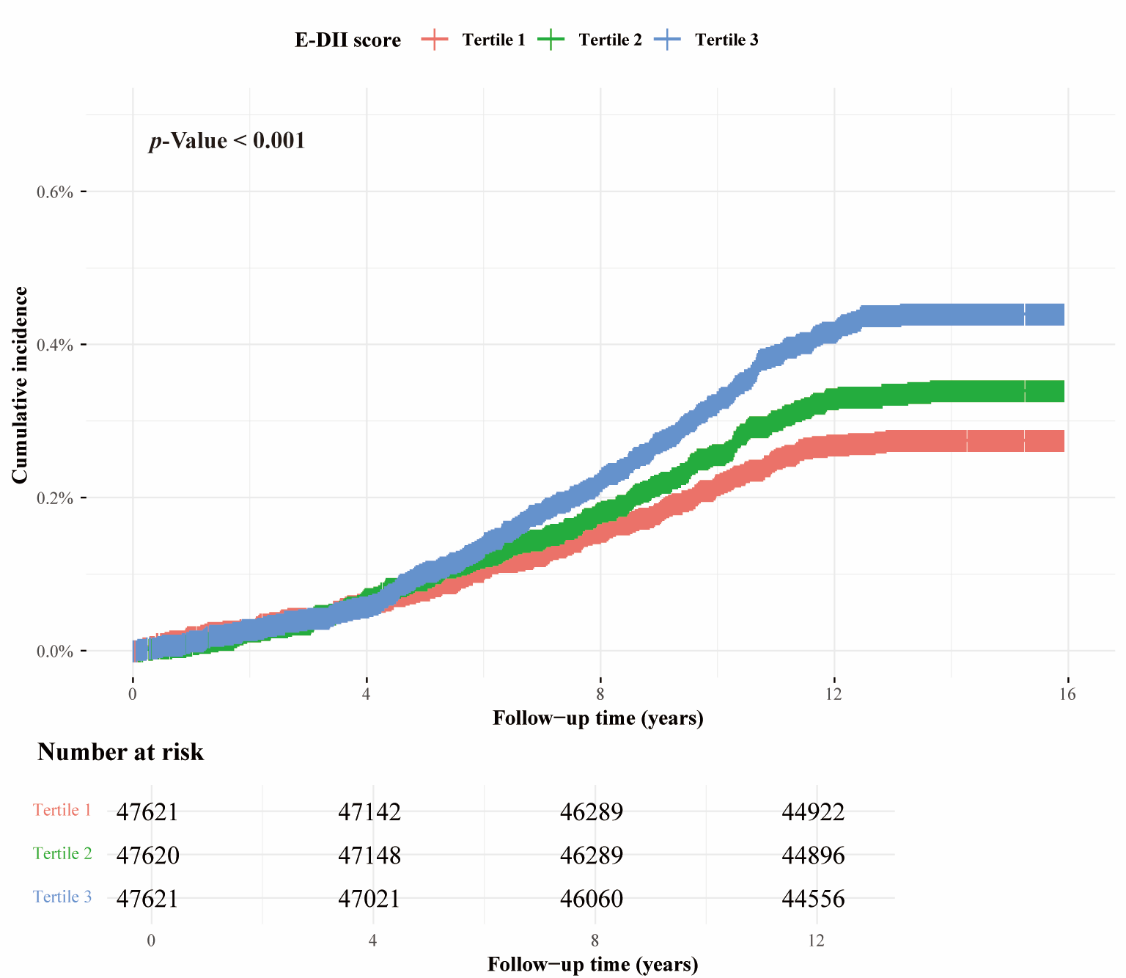


**Supplementary Figure S3.** Density plot of the E-DII and identified threshold


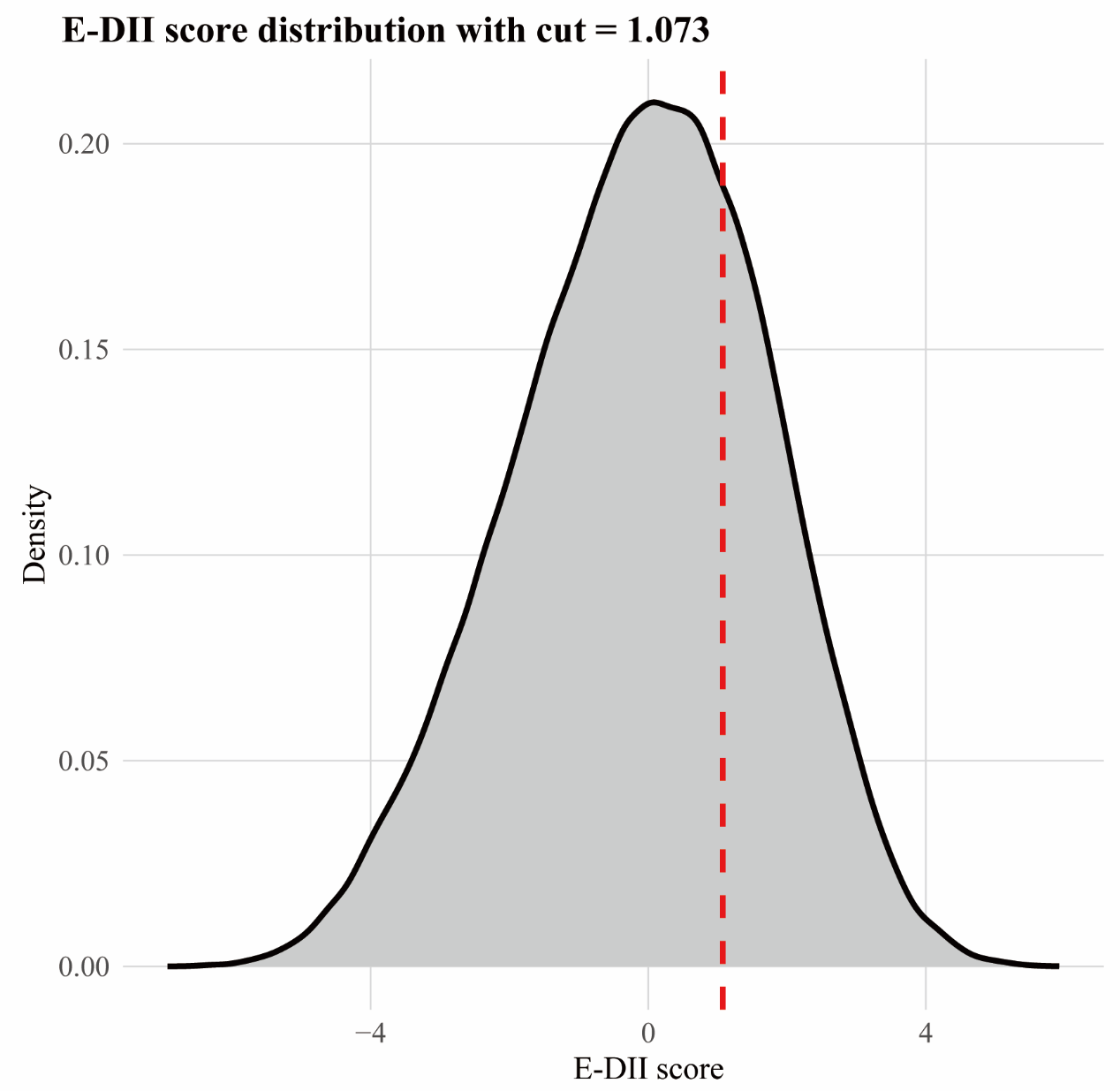


The distribution of the E-DII score determined by the maximally selected rank statistics. The optimal cut-off value is indicated by the vertical dashed line. The y-axis represents probability density, and the x-axis represents the observed E-DII scores.

**Supplementary Figure S4.** Receiver Operating Characteristic Curves for Three Polygenic Risk Scores


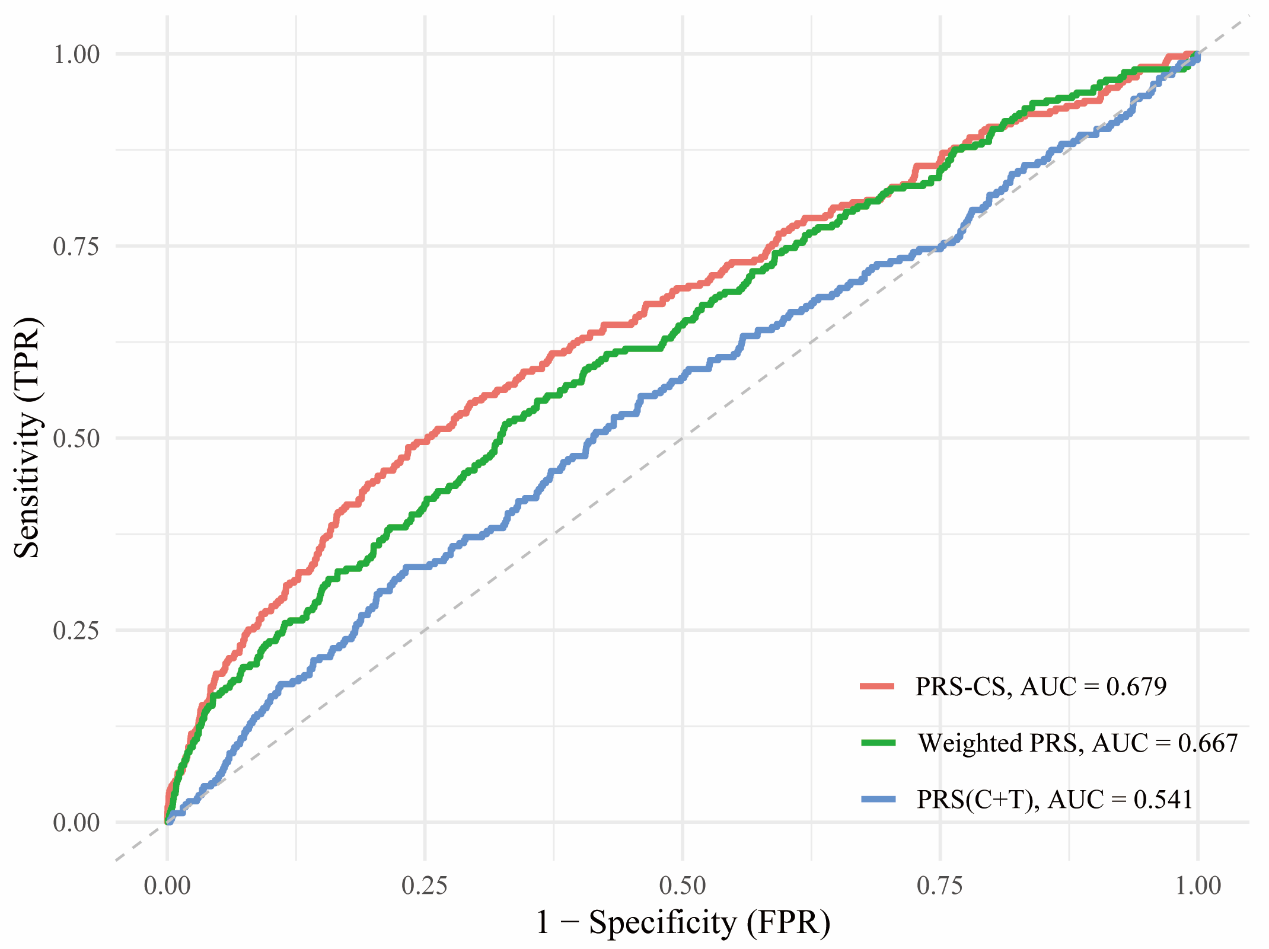


Abbreviations: PRS, Polygenic risk score; TPR, True Positive Rate; FPR, False Positive Rate; AUC, Area Under the Curve.

**Supplementary Figure S5.** Interaction between the E-DII score and polygenic risk score in relation to AAA risk


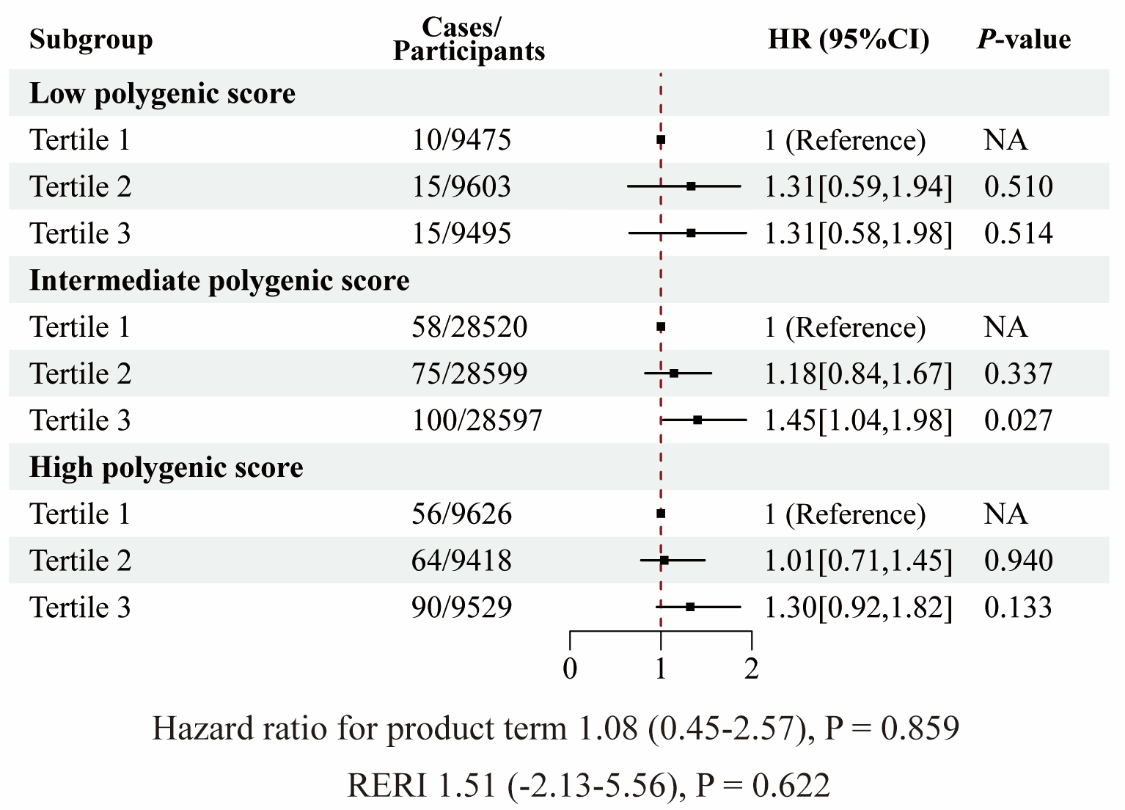


Analyzes were adjusted for sex, age, education level, employment status, Townsend Deprivation Index, body mass index, prior medication use, vitamin supplementation, medical history (encompassing hypertension, diabetes, dyslipidemia, cardiovascular and cerebrovascular diseases, cancer, chronic respiratory diseases, chronic kidney disease, and chronic liver disease), smoking history, physical activity, sleep pattern, and sedentary time.
